# Research hotspots and trends in post-stroke dysphagia: a bibliometric analysis

**DOI:** 10.3389/fnins.2023.1275748

**Published:** 2023-10-24

**Authors:** Fangyuan Xu, Lin Bai, Ziliang Dai, Hongliang Cheng

**Affiliations:** ^1^The First Clinical Medical School, Anhui University of Chinese Medicine, Hefei, China; ^2^Department of Neurology, The Second Affiliated Hospital of Anhui University of Chinese Medicine, Hefei, China; ^3^Department of Rehabilitation Medicine, Wuhan Iron and Steel (Group) Second Staff Hospital, Wuhan, China

**Keywords:** post-stroke dysphagia, stroke, bibliometric analysis, CiteSpace, VOSviewer

## Abstract

**Background:**

Dysphagia represents one of the common complications following a stroke, and post-stroke dysphagia (PSD) can lead to aspiration, pneumonia, and malnutrition, thus prolonging hospital stay, escalating medical expenditures, and imposing a substantial economic strain on both patients and society. The utilization of bibliometric analysis offers a quantitative approach for investigating the existing literature and recognizes the current status of the research. However, bibliometric analysis on the subject of PSD remains absent. Consequently, we carried out this study to provide researchers with insights, facilitating their further exploration of PSD.

**Methods:**

Conducting a bibliometric analysis of articles pertaining to PSD retrieved over the past two decades enables us to acquire the research hotspots and trends in this area. The publications concerning PSD were searched from the Core Collection of Web of Science, spanning the period ranging from 2003 to 2023. Articles or reviews published in English were included in this study. Subsequently, we employed CiteSpace and VOSviewer software to visualize the retrieved articles, thereby identifying the cooperative relationships of authors, institutions, and countries, as well as relevant information about journals and references.

**Results:**

This study comprised 866 papers in total, and the number of articles published each year shows an overall growth trend. As for the analysis of the authors, Dziewas R. was the most prolific author with 21 articles. The most frequently published institutions, countries, and journals were the University of Manchester, China, and *Dysphagia*, with 28, 254, and 75 publications, respectively. And the co-cited authors and journals with the highest counts were Martino R and *Stroke*. According to the analysis of keywords and references, dysphagia screening and assessment, prevention of pneumonia, rehabilitation approaches, and nutritional management of PSD are considered research hotpots. Additionally, future research may focus on the topics of systematic review and meta-analysis, noninvasive brain stimulation, and lesion location.

**Conclusion:**

Through the bibliometrics analysis of PSD, we can capture the research hotspots and frontiers of PSD, thereby providing inspiration and reference for subsequent studies in this field.

## 1. Introduction

The process of swallowing involves the coordination of multiple muscle groups and nerves to facilitate transporting food or liquids from the oral cavity to the stomach (Jones et al., 2020). Oropharyngeal and esophageal dysphagia can be distinguished based on the distinct phases of the swallowing process, with stroke emerging as a significant contributing factor to oropharyngeal dysphagia (Wilkinson et al., 2021). Post-stroke dysphagia (PSD), characterized by impaired swallowing function following a stroke, is a common stroke-associated complication. Due to the differences between countries and the accuracy of measurement techniques, previous studies reported that the prevalence of PSD ranged from 37 to 81% (Meng et al., 2000; Martino et al., 2005; Ko et al., 2021). While there is spontaneous improvement in swallowing disorders among many stroke patients, it was shown that 11–50% of individuals remained to have dysphagia six months after the stroke (Smithard et al., 1997; Mann et al., 1999). The higher risk of aspiration, pneumonia, and malnutrition is correlated with PSD, thereby prolonging hospital stays, increasing medical expenses, and imposing a significant economic burden on individuals and society (Cohen et al., 2016). In conclusion, PSD influences the prognosis and quality of life of stroke patients and causes substantial financial burden, which is worthy of extensive public attention.

Due to the high prevalence of PSD, it is necessary to conduct dysphagia screening by assessing the swallowing function of hospitalized stroke patients. Furthermore, determining the occurrence and severity of PSD makes it possible to detect the risk of aspiration (Fang et al., 2022). The evaluation of dysphagia comprises bedside screening, clinical swallowing assessment, as well as further instrumental assessment like fiberoptic endoscopic evaluation of swallowing (FEES) or videofluorographic swallowing study (VFSS) (Re et al., 2018; Helliwell et al., 2023). The primary approaches for managing PSD incorporate compensatory techniques, behavioral interventions, biofeedback, acupuncture therapy, and peripheral and central neurostimulation methods such as neuromuscular electrical stimulation (NMES) and repetitive transcranial magnetic stimulation (rTMS) (Wang et al., 2017; Bath et al., 2018; Archer et al., 2021; Lu et al., 2021). Despite the emergence and rapid development of numerous management approaches for PSD, there still lacks bibliometric analysis to investigate the research status, hotspots, and future research tendencies in this topic. Therefore, a bibliometric analysis pertaining to PSD is urgently required to thoroughly synthesize the research emphasis and provide valuable information for clinical practice and the advancement of future studies.

The bibliometric analysis provides a quantitative approach for the comprehensive exploration of the existing literature within a certain research area. This approach involves an in-depth analysis of information extracted from the literature, encompassing authors, institutions, countries, journals, keywords, and references (Ellegaard and Wallin, 2015; Merigó et al., 2015). It serves to discern the current development status and emerging research directions. Furthermore, the results are displayed in network visualization maps, which offer a clearer and more intuitive perspective for comprehending the collaborative relationships within the field. Consequently, we employed CiteSpace and VOSviewer software to conduct a bibliometric and visual analysis of articles addressing PSD in the past two decades.

## 2. Materials and methods

### 2.1. Literature source and data retrieval methods

**Figure 1:**
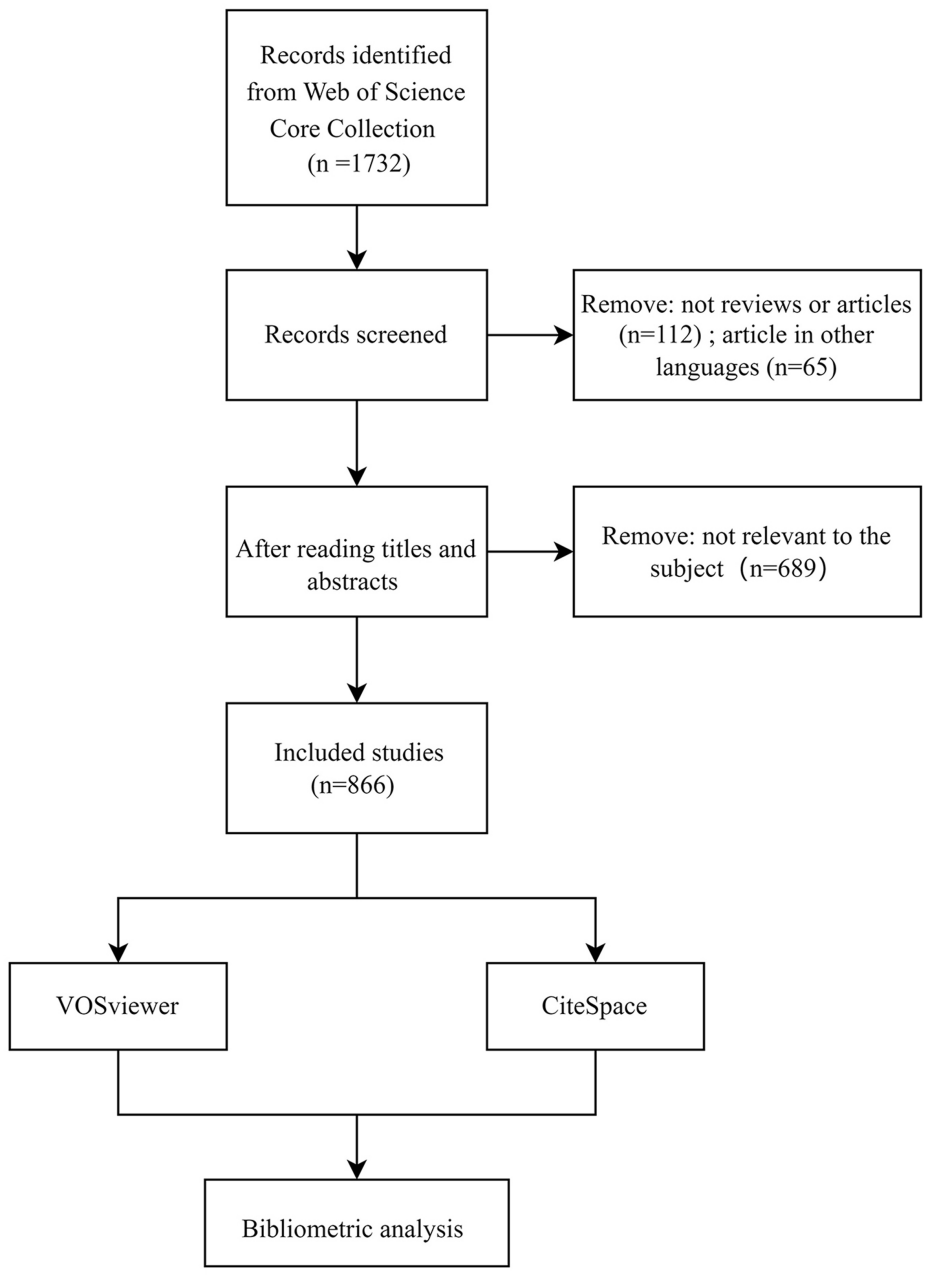
The flow chart for screening the included literature.

We performed a comprehensive retrieval utilizing the core collection of the Web of Science database to identify studies pertinent to PSD on July 16, 2023. The search formula was presented as follows: TS = (post-stroke dysphagia OR dysphagia after stroke OR post-stroke deglutition disorder OR deglutition disorder after stroke OR post-stroke swallowing disorder OR post-stroke swallowing dysfunction OR swallowing disorder after stroke). The time span was 2003–2023, and only articles and reviews were included in this study. Additionally, the language was limited to English. Exclusion criteria were unavailable or incomplete studies, duplicated studies, and literature irrelevant to the subject. The flow chart for screening the included literature is presented in Figure 1. Upon completing the literature retrieval, the selected publications were exported with full records in TXT form and prepared for subsequent bibliometric analysis.

### 2.2. Data analysis

The bibliometric analysis was conducted using CiteSpace V6.2.R3 and VOSviewer V1.6.19 tools, which were based on Java. VOSviewer (van Eck and Waltman, 2010) was used to conduct cooperation or co-occurrence networks of authors, countries, institutions, journals, and keywords. The size of a node indicates its frequency of occurrence, while the connections between nodes depict its co-occurrence or collaboration relationship. The thicker the lines represent, the stronger the intensity of the association is. In addition, the colors of nodes and links manifest different clusters (Wang et al., 2022; Huang et al., 2023). CiteSpace (Chen, 2006) was applied to generate the co-occurrence and clustering map of reference. In the meantime, we displayed keywords and references with strong citation bursts to capture the emerging tendencies. When analyzing the study results, betweenness centrality serves as an indicator for evaluating the significance of nodes in the visualization network. Nodes with high centrality, more than 0.1, are regarded as crucial points and are visually distinguished by a purple circle outside the respective nodes.

## 3. Results

### 3.1. Publication trends

**Figure 2:**
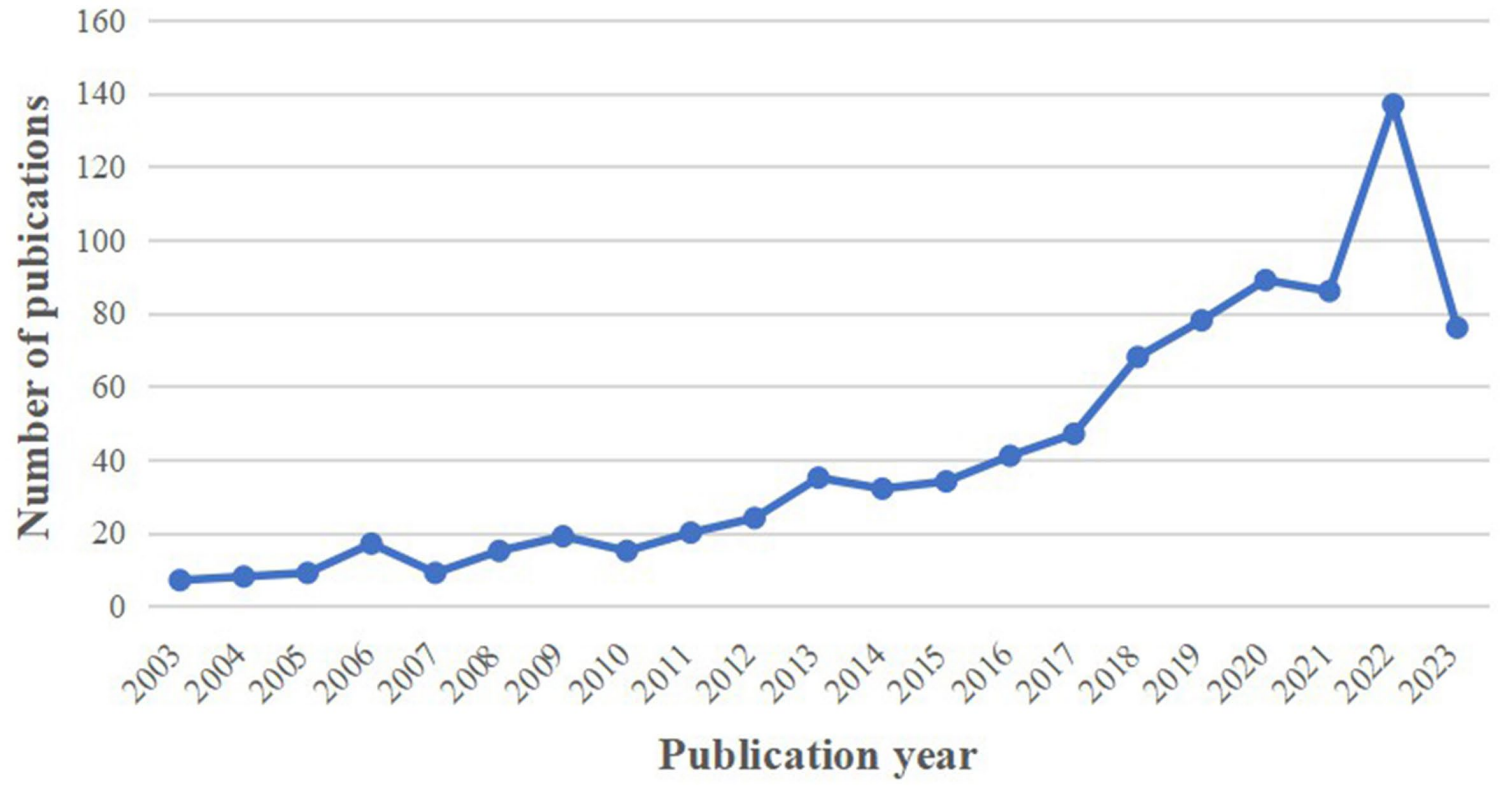
Annual number and trend of publications.

This bibliometric analysis covered 866 papers pertaining to the topic of PSD, comprising 746 articles (86.14%) and 120 reviews (13.86%). As depicted in Figure 2, while there were fluctuations over the years, the annual publication number exhibited an overall upward trend. Notably, there was a marked growth in publications in 2018 and 2022 compared to the previous year. The peak was reached in 2022 with 137 articles, and 76 papers were published in 2023 as of the retrieval date. The above trend suggests a growing emphasis by scholars on the subject of PSD, and they are expected to carry out more articles promoting the development of PSD.

### 3.2. Analysis of authors

The 866 retrieved articles included a total of 4,213 authors. According to Price’s Law, the minimum publication volume for core authors is *m* = 0.749× $\surd n_{\max}$ . The *n*_max_ indicates the volume of papers produced by the most prolific authors (*n*_max_ = 21 here). Therefore, the core authors of PSD studies should have published more than four papers. Price put forward that half of the papers in the same domain were accomplished by highly productive authors (Price, 1963). In line with Price’s theory, our analysis identified 106 core authors who published 624 articles, constituting 72.06% of the total literature.

**Figure 3:**
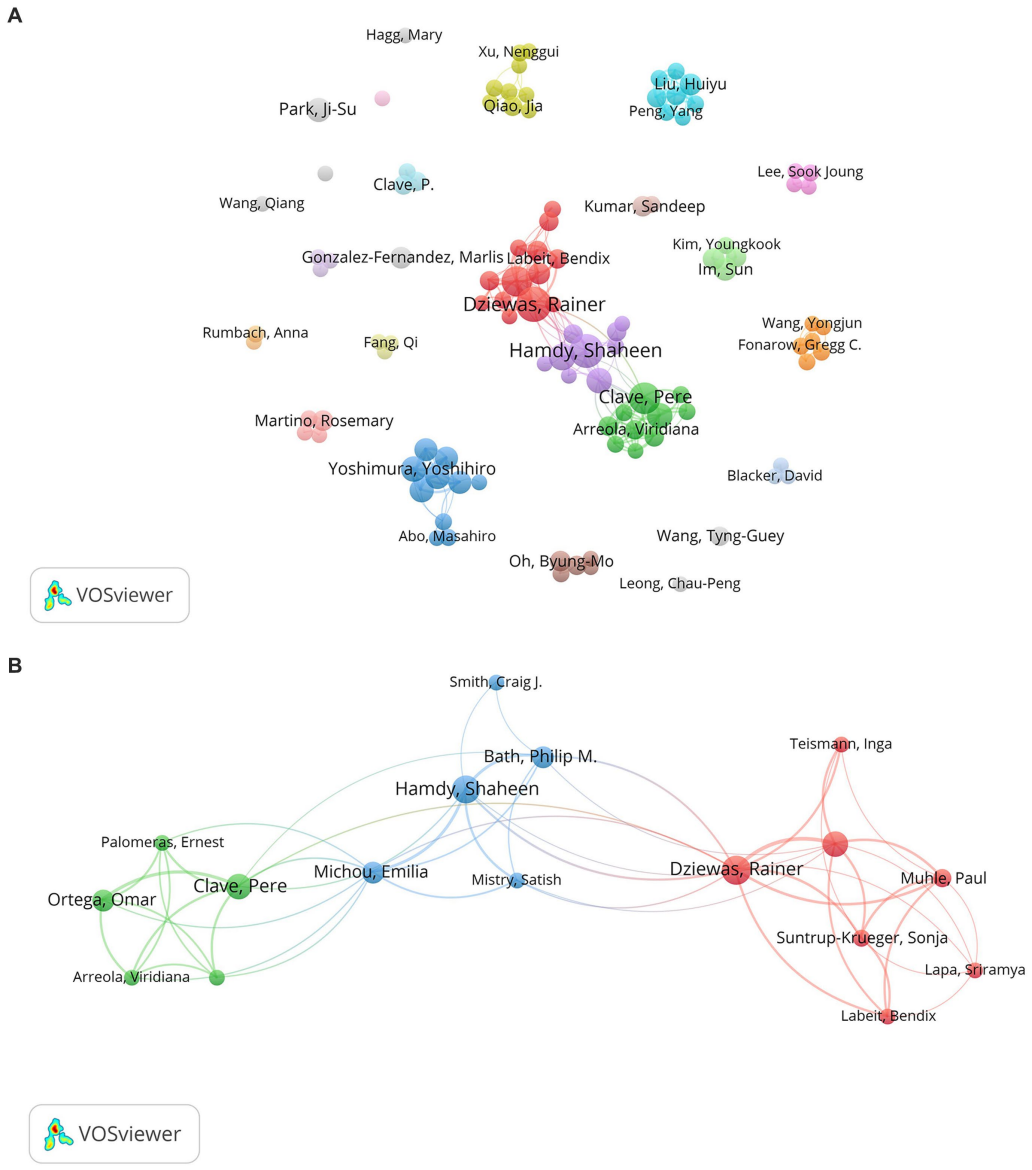
**(A)** The collaboration network map of core authors about PSD; **(B)** The main three clusters of core authors.

To display the cooperation relationship among core authors, the visualization map generated by the VOSviewer is shown in Figure 3A. Furthermore, Figure 3B presents the three main clusters of core authors. These figures reveal that several stable groups of core authors have been formed, while there remains potential for further collaboration between different groups. As depicted in Table 1, Dziewas R. was the most prolific author among those core authors, having published 21 articles from 2004 to 2010 with 681 citations. Dziewas R. concentrated on dysphagia screening and evaluation, the cortical swallowing process in stroke patients, the occurrence and risk factors of pneumonia, and the efficacy of treatment such as pharyngeal electrical stimulation (PES) (Dziewas et al., 2004; Warnecke et al., 2009; Teismann et al., 2011; Suntrup et al., 2015; Warnecke et al., 2017). Among the average citations of the authors, Bath P. M. ranks first (approximately 45 times), indicating that articles written by Bath P. M. are influential. The emphasis of his research was mainly concerning transcranial magnetic stimulation, PES, or other swallowing therapies for improving PSD (Bath et al., 2018; Sasegbon et al., 2019; Everton et al., 2021).

**Table 1:** The top 10 core authors with the most publications.

Rank	Author	Documents	Citations	Average citation
1	Dziewas R	21	681	32
2	Hamdy S	19	813	43
3	Clave P	16	277	17
4	Warnecke T	15	530	35
5	Bath PM	12	536	45
6	Ortega O	11	141	13
7	Yoshimura Y	11	230	21
8	Michou E	11	406	37
9	Nagano F	10	223	22
10	Shimazu S	10	223	22

### 3.3. Analysis of countries and institutions

**Figure 4:**
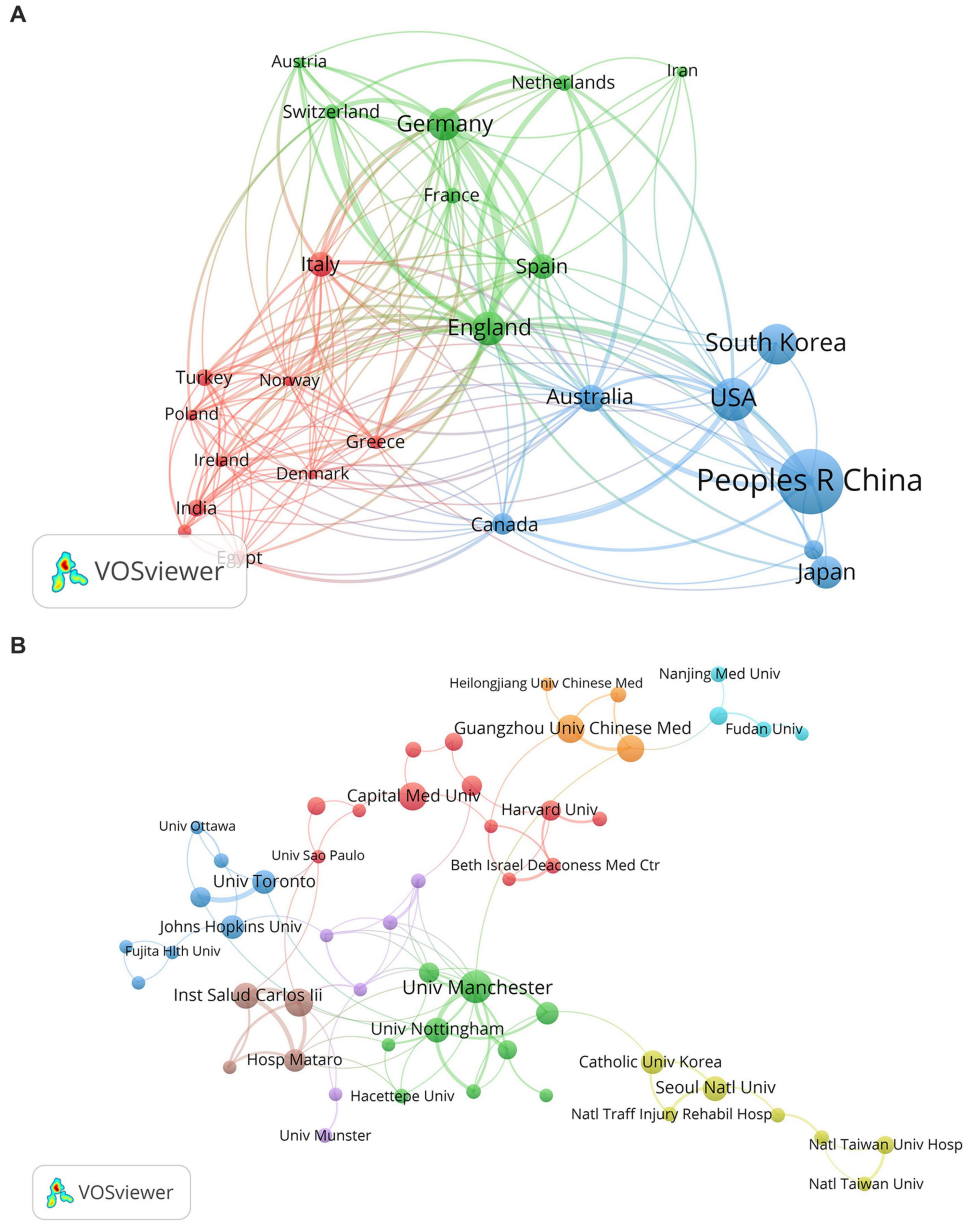
**(A)** The cooperation network map of countries about PSD; **(B)** The cooperation network map of institutions about PSD.

Through the analysis of published literature, we can identify countries that have demonstrated more interest in PSD and have exerted great efforts in the development of this field. Fifty-eight countries participated in the studies of PSD from 2003 to 2023, and the cooperation map of countries was conducted using VOSviewer. Figure 4A shows the main cooperation network map of nations, where the link strength manifests the intensity of cooperation between nations. England, Germany, and Australia had the greatest total link strength, with 88, 59, and 44 respectively. This indicates that the three countries maintained close associations with other countries. According to the data presented in Table 2, China was the most prolific country with 254 articles, followed by the USA (111 papers) and South Korea (100 articles). And studies from these three countries accounted for 53.7% of the total 866 articles. It deserved attention that China had the highest publication count, but its international collaborations appeared not to be close enough. Therefore, it is necessary for China to strengthen global communication and cooperation in PSD research.

**Table 2:** The top 10 countries and institutions in terms of frequency.

Rank	Country	Frequency	Total link strength	Institution	Frequency	Total link strength
1	Peoples R China	254	37	University of Manchester	28	39
2	USA	111	42	Autonomous University of Barcelona	20	29
3	South Korea	100	7	Guangzhou University of Chinese Medicine	20	10
4	England	70	88	Capital Medical University	20	3
5	Germany	64	59	Sun Yat-sen University	19	10
6	Japan	63	7	Instituto de Salud Carlos III	17	26
7	Australia	43	44	University of Nottingham	16	27
8	Spain	38	40	Seoul National University	16	10
9	Italy	36	37	University of Toronto	15	14
10	Canada	28	28	The Catholic University of Korea	14	7

This study analyzed 866 publications from 1,316 institutions. Table 2 shows the top 10 institutions with publications in PSD. The University of Manchester had the most considerable output (28 articles), followed by the Autonomous University of Barcelona, Guangzhou University of Chinese Medicine, and Capital Medical University with 20 papers. Besides, the University of Manchester, the Autonomous University of Barcelona, and the University of Nottingham have the highest total link strength (39, 29, 27, respectively), indicating their close collaboration with other institutions. Figure 4B mainly displays institutions that have published a minimum of five articles. The clusters are relatively scattered, which proves that collaboration between institutions requires strengthening.

### 3.4. Analysis of journals and co-cited journals

**Figure 5:**
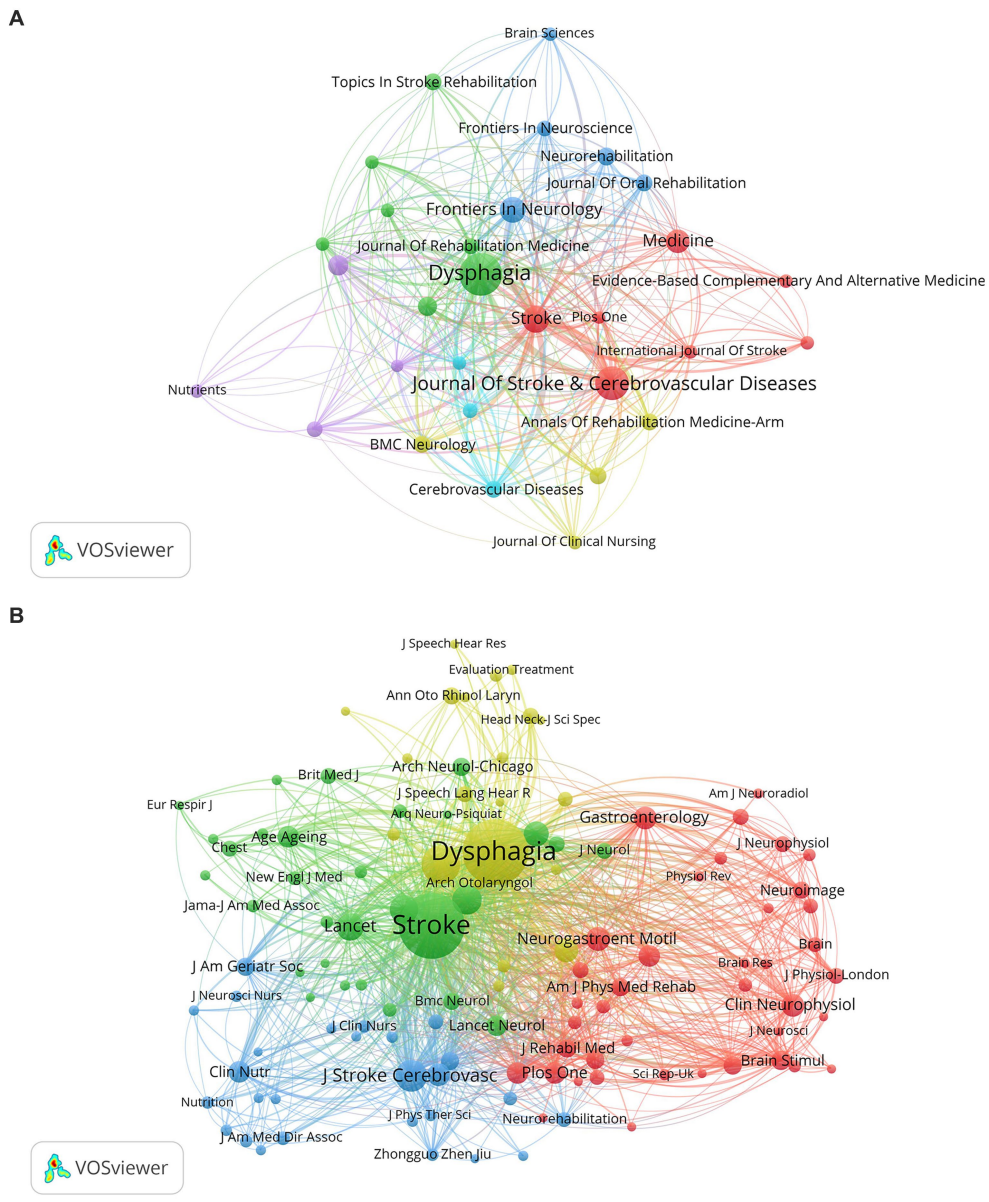
**(A)** The network visualization map of journals about PSD; **(B)** The visualization map of co-cited journals about PSD.

The included papers are distributed in different journals, and the top 10 journals with the highest frequency are listed in Table 3. The journals that published the most PSD articles were *Dysphagia* (75 articles), *Journal of Stroke & Cerebrovascular Diseases* (46 articles), and *Stroke* (30 articles), among which *Stroke* had the highest impact factor in 2022. There are only two of the top 10 journals were in the Q1 partition. We generated a visualization map of journals with at least five articles and displayed it in Figure 5A. The map reveals that *Stroke*, *Dysphagia*, and the *Journal of Stroke & Cerebrovascular Diseases* occupy center positions, representing close associations with other journals distributed in different clusters.

**Table 3:** The top 10 journals and co-cited journals with the most frequency or citations.

Rank	Journal	Frequency	2022 JCR (IF)	Co-cited journal	Citations	2022 JCR (IF)
1	Dysphagia	75	Q2 (2.6)	Stroke	3,078	Q1 (8.3)
2	Journal of Stroke & Cerebrovascular Diseases	46	Q3 (2.5)	Dysphagia	3,067	Q2 (2.6)
3	Stroke	30	Q1 (8.3)	Archives of Physical Medicine and Rehabilitation	946	Q1 (4.3)
4	Frontiers in Neurology	26	Q2 (3.4)	Journal of Stroke & Cerebrovascular Diseases	636	Q3 (2.5)
5	Medicine	22	Q3 (1.6)	Neurology	543	Q1 (9.9)
6	Neurogastroenterology and Motility	16	Q2 (3.5)	Cerebrovascular Diseases	480	Q3 (2.9)
7	Archives of Physical Medicine and Rehabilitation	15	Q1 (4.3)	Lancet	425	Q1 (168.9)
8	Neurorehabilitation	13	Q2 (2)	Journal of Neurology, Neurosurgery and Psychiatry	381	Q1 (11)
9	Cerebrovascular Diseases	12	Q3 (2.9)	Neurogastroenterology and Motility	339	Q2 (3.5)
10	Journal of Oral Rehabilitation	12	Q2 (2.9)	Clinical Neurophysiology	308	Q1 (4.7)

As for co-cited journals, Figure 5B presents the network visualization map with four clusters in different colors. The largest red cluster indicates that journals mainly concentrate on the subjects of brain science, neurology, and rehabilitation. The green, blue, and yellow clusters are headed by *Stroke*, *Journal of Stroke & Cerebrovascular Diseases*, and *Dysphagia*, respectively. According to the results shown in Table 3, the most frequently co-cited journals were *Stroke* (3,078 citation times), followed by *Dysphagia* (3,067 times), and *Archives of Physical Medicine and Rehabilitation* (946 times). Among the top 10 co-cited journals, the 2022 impact factors ranged from 2.5 to 168.9, and more than half of the journals were categorized as Q1 partitions.

### 3.5. Analysis for co-occurrence and citation burst of keywords

**Figure 6:**
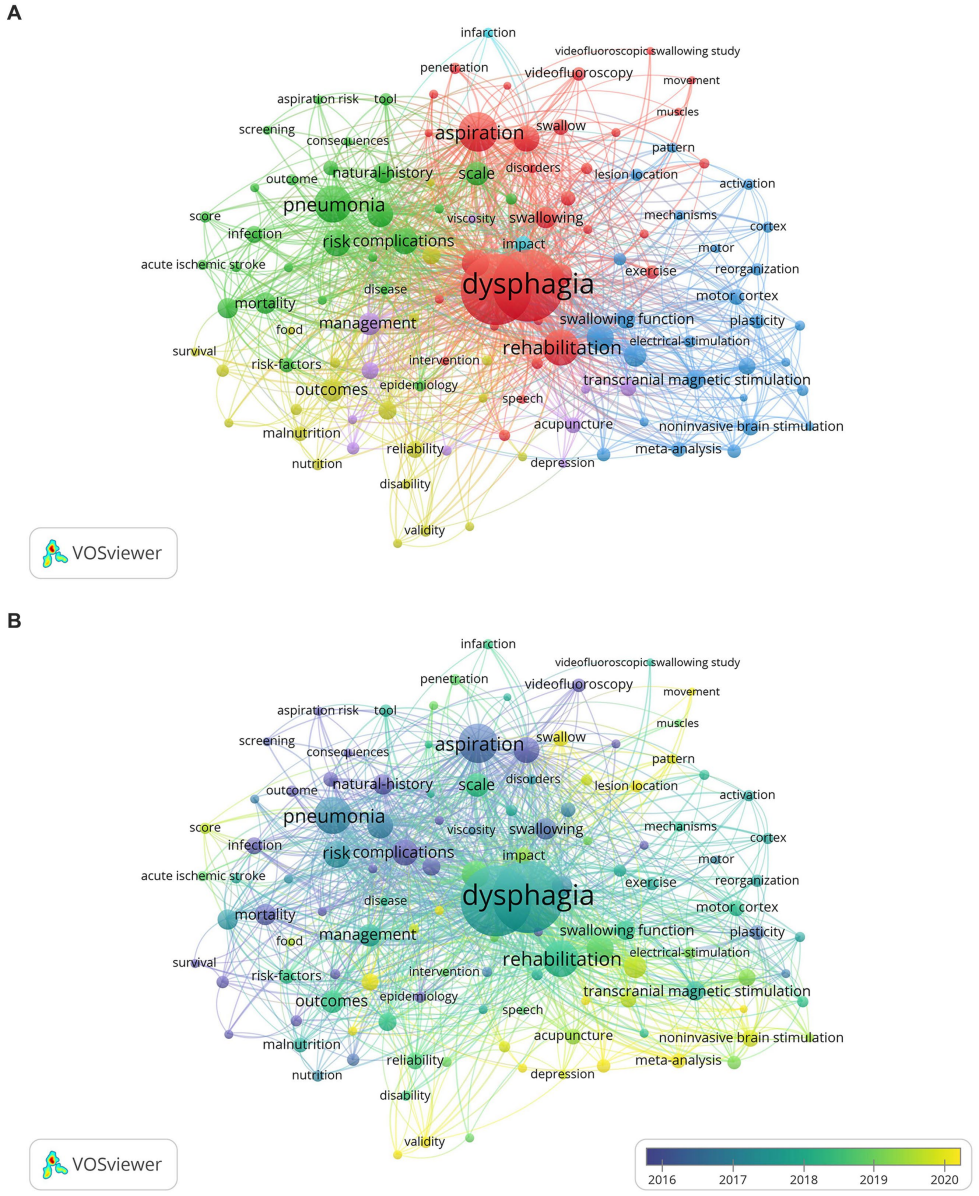
**(A)** The co-occurrence and clustering map of keywords about PSD; **(B)** The overlap visualization map of keywords about PSD.

Keywords serve as concise generalizations of article content. In Figure 6A, we display the co-occurrence and clustering map of keywords generated using VOSviewer, which have been classified into four prominent clusters. The red cluster primarily addresses the rehabilitation and assessment of PSD, while the green cluster focuses on the risk of aspiration and pneumonia. The yellow cluster mainly concerns malnutrition and nutritional management, and the blue cluster is about neurostimulation therapy for improving swallowing function. As listed in Table 4, the most frequently occurring keywords are dysphagia, stroke, aspiration, pneumonia, and rehabilitation, with 671, 650, 200, 182, and 176 times, respectively. And dysphagia also exhibits the highest total link strength (4,182) among them. The overlay visualization map of keywords shown in Figure 6B reflects the changes in research trends over time. Purple and blue nodes correspond to keywords that emerged earlier, indicating that scholars performed extensive research on mortality, prognosis, complications, aspiration, and pneumonia concerning PSD. Keywords in green and yellow appeared later, suggesting the subsequent research emphasis on various dysphagia rehabilitation approaches, cortex, and lesion location.

**Table 4:** The top 10 keywords in terms of frequency related to PSD.

Rank	Keyword	Frequency	Total link strength
1	Dysphagia	671	4,182
2	Stroke	650	4,103
3	Aspiration	200	1,400
4	Pneumonia	182	1,302
5	Rehabilitation	176	1,331
6	Risk	99	738
7	Diagnosis	98	735
8	Recovery	96	761
9	Predictors	91	685
10	Deglutition	89	651

**Figure 7:**
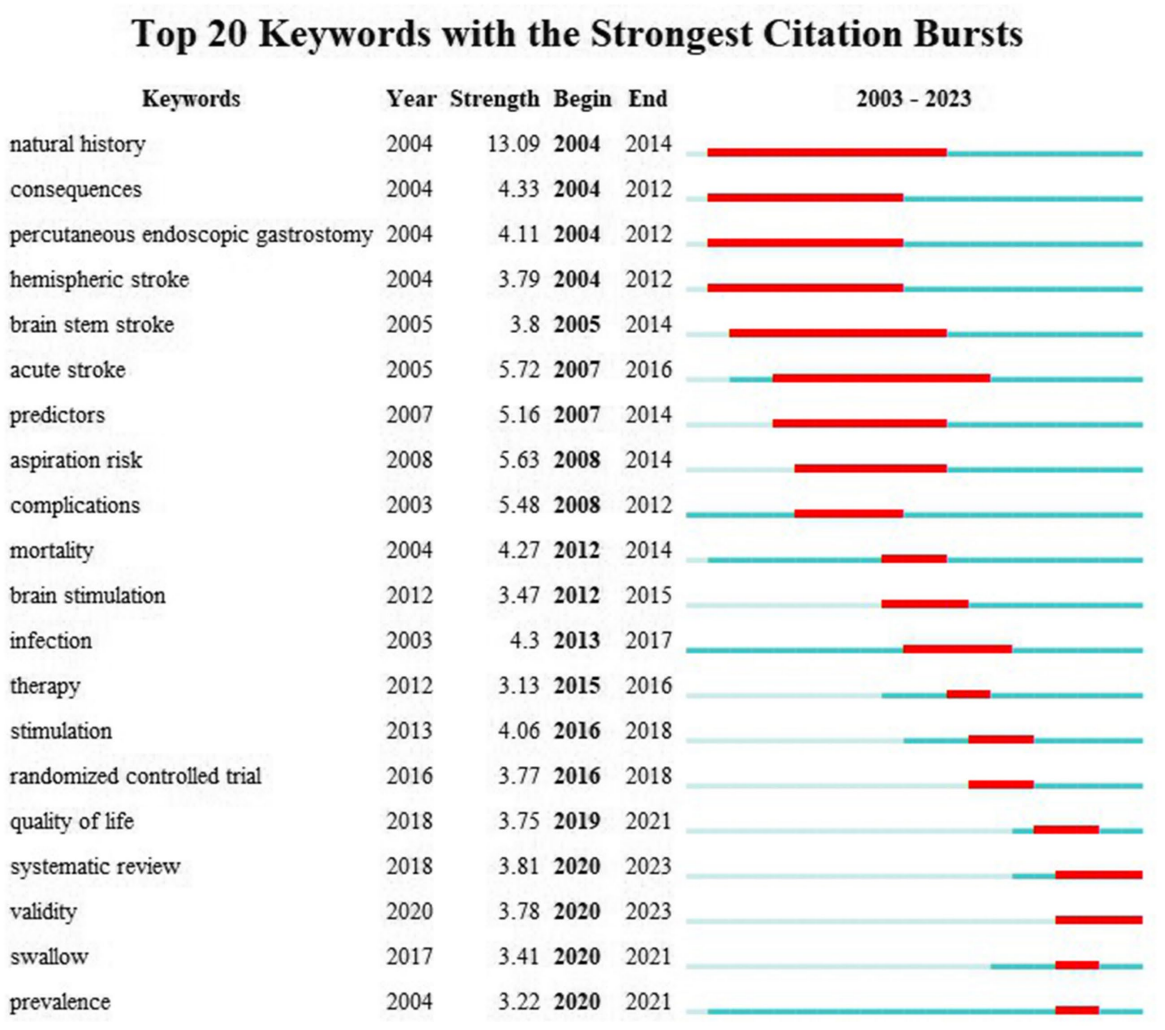
Top 20 keywords with the strongest citation bursts from 2003 to 2023.

The keyword burst refers to a sharp increase in keyword occurrences within a certain period of time, which serves as valuable indicators for acquiring research emphasis and frontiers (Hu et al., 2022). Burst strength and time span are vital indicators in keyword burst analysis. According to the results of Figure 7, the top three keywords with the strongest burst strength are “natural history” (2004–2014), “acute stroke” (2007–2016), and “aspiration risk” (2008–2014). “Systematic review” and “validity” are two keywords with strong citation bursts recently, and this tendency may persist in the upcoming years.

### 3.6. Analysis of co-cited authors

**Figure 8:**
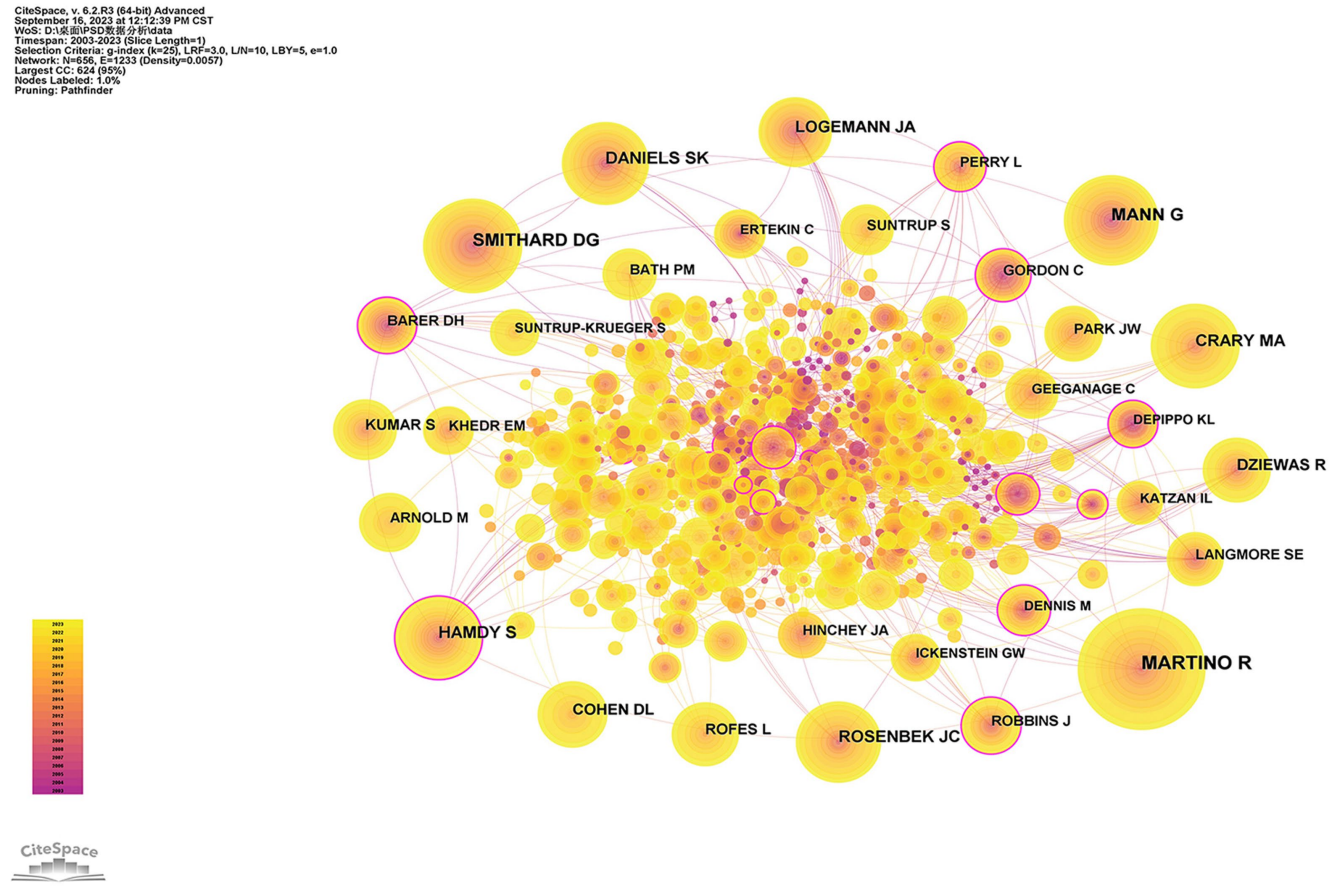
The visualization map of co-cited authors about PSD.

The analysis of co-cited authors may provide a more thorough understanding of influential authors who are widely recognized by scholars in the research field. The network map of co-cited authors is displayed in Figure 8. Furthermore, Table 5 lists the top 10 co-cited authors with the most frequency and centrality. Martino R received the highest number of citations (387 times). His most frequently cited article elucidated that the risk of acquiring pneumonia would be increased in patients with PSD, and the risk became even greater for patients with confirmed aspiration (Martino et al., 2005). Smithard D. G. ranks as the second co-cited author in our analysis. His study demonstrated that dysphagia was more likely to occur in those who experienced a total anterior cerebral infarction. Additionally, it was shown that dysphagia in the acute phase of stroke was related to increased mortality, particularly during the first 3 months. However, this relationship gradually decreased over the five-years follow-up period (Smithard et al., 2007). Fraser C had the highest centrality of 0.18. His most frequently cited article illustrated that the excitability of the corticobulbar projection to the pharynx depended on the frequency, intensity, and duration of the electrical pharyngeal stimulation. Moreover, the application of such stimulation can enhance sensorimotor cortex nerve function and lead to the improvement of swallowing capabilities (Fraser et al., 2002).

**Table 5:** The top 10 co-cited authors in terms of frequency and centrality.

Rank	Co-cited author	Frequency	Co-cited author	Centrality
1	Martino R	387	Fraser C	0.18
2	Smithard DG	264	Dennis M	0.17
3	Mann G	222	Freed ML	0.16
4	Daniels SK	190	Perry L	0.15
5	Hamdy S	187	Foley N	0.15
6	Crary MA	183	Robbins J	0.14
7	Rosenbek JC	173	Depippo KL	0.14
8	Logemann JA	150	Kidd D	0.14
9	Dziemas R	122	Davenport RJ	0.14
10	Cohen DL	110	Hamdy S	0.13

### 3.7. Analysis of co-occurrence, clustering, and citation burst of references

**Figure 9:**
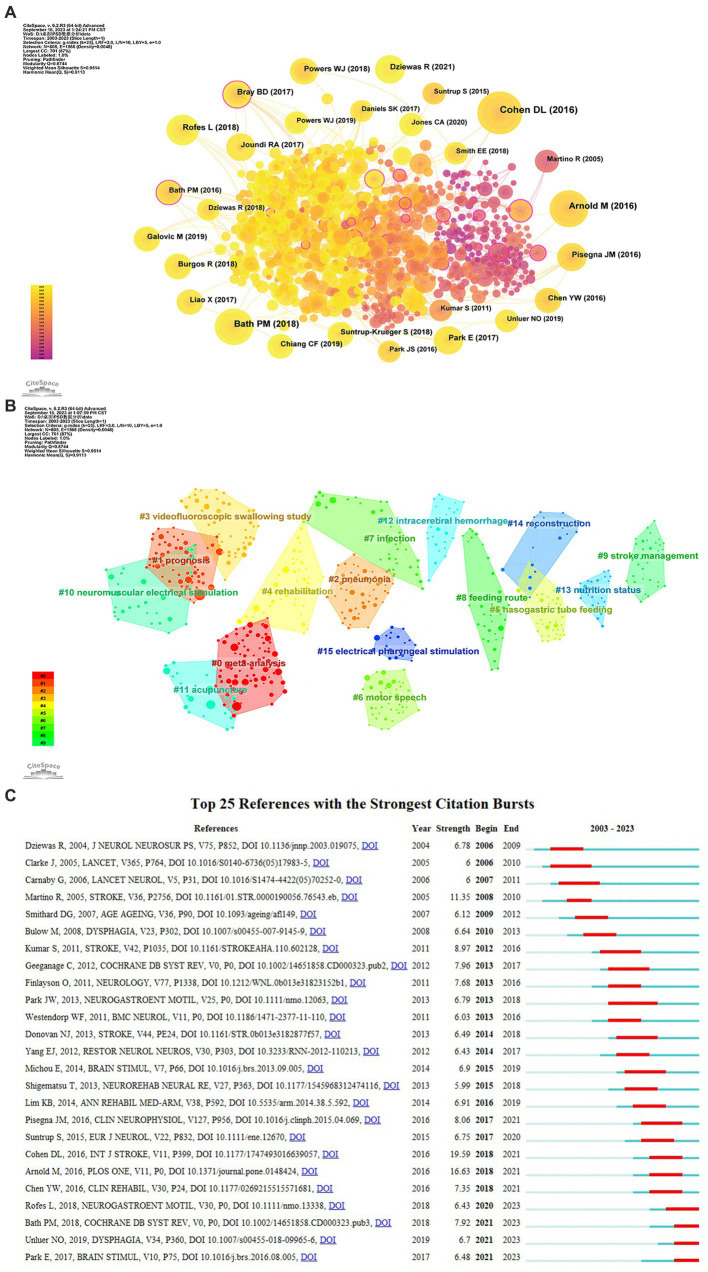
**(A)** The co-occurrence map of co-cited references about PSD; **(B)** The clustering map of co-cited references; **(C)** Top 25 references with the strongest citation bursts from 2003 to 2023.

Co-citation analysis is conducive to identifying the knowledge base and influential articles from an enormous amount of references, enabling a deeper exploration of the development in this field. As shown in Figure 9A, the yellow and orange nodes occupy a large portion of the co-cited references map, suggesting that recent literature has gained more frequent citations. Table 6 shows the top 10 co-cited references pertinent to PSD, comprising five clinical studies, a review, two meta-analyses, and two guidelines. The most frequently cited and influential article, conducted by Cohen et al. (2016), also exhibits the most prominent reference burst strength (see Figure 9C). This article provides a comprehensive overview of the epidemiology, pathogenesis, complications, diagnosis, management, and treatment of PSD. Furthermore, Cohen emphasized the importance of oral care from the patient’s perspective and called for more rigorous clinical study designs from the researchers’ standpoint (Cohen et al., 2016). Rofes et al. (2018) and Arnold et al. (2016) explored the incidence of PSD and indicated that dysphagia was a risk factor for poor prognosis and increased mortality within 3 months post-stroke. However, it is important to consider the potential presence of selection bias in the included patients, which could impact the interpretation of the prevalence, risk factors, and clinical outcomes of PSD. In a systematic review, Bath et al. (2018) comprehensively synthesized the effects of eight swallowing therapies on PSD, indicating that these interventions may enhance the function of deglutition with very low-quality evidence. Consequently, more rigorously designed clinical trials are needed to validate and compare the effectiveness and safety of different therapies.

**Table 6:** The top 10 co-cited references related to PSD.

Rank	Counts	Co-cited reference	References
1	63	Post-stroke dysphagia: A review and design considerations for future trials	Cohen et al. (2016)
2	47	Dysphagia in Acute Stroke: Incidence, Burden and Impact on Clinical Outcome	Arnold et al. (2016)
3	46	Swallowing therapy for dysphagia in acute and subacute stroke	Bath et al. (2018)
4	32	Prevalence, risk factors and complications of oropharyngeal dysphagia in stroke patients: A cohort study	Rofes et al. (2018)
5	27	Effects of Bilateral Repetitive Transcranial Magnetic Stimulation on Post-Stroke Dysphagia	Park et al. (2017)
6	27	European Stroke Organisation and European Society for Swallowing Disorders guideline for the diagnosis and treatment of post-stroke dysphagia	Dziewas et al. (2021)
7	27	Effects of non-invasive brain stimulation on post-stroke dysphagia: A systematic review and meta-analysis of randomized controlled trials	Pisegna et al. (2016)
8	26	The association between delays in screening for and assessing dysphagia after acute stroke, and the risk of stroke-associated pneumonia	Bray et al. (2017)
9	25	Predictors and outcomes of dysphagia screening after acute ischemic stroke	Joundi et al. (2017)
10	24	2018 Guidelines for the Early Management of Patients With Acute Ischemic Stroke: A Guideline for Healthcare Professionals From the American Heart Association/American Stroke Association	Powers et al. (2018)

Pisegna et al. (2016) showed evidence for non-invasive brain stimulation in the promotion of PSD recovery, but differences in stroke types, severity, and outcome indicators across the included literature may result in heterogeneity. Park et al. (2017) indicated that 10-Hz rTMS at the bilateral cortical area projecting to the mylohyoid muscle had better cumulative effects in enhancing swallowing ability than the ipsilesional rTMS. However, the sample size of this study is relatively small; further studies can expand the sample size to verify the reliability of the conclusions. The two guidelines provide valuable guidance for clinical decision-making and point out further research directions in the treatment and management of PSD (Powers et al., 2018; Dziewas et al., 2021). Moreover, Joundi et al. (2017) and Bray et al. (2017) placed emphasis on dysphagia screening after acute stroke and indicated a higher pneumonia risk related to delayed dysphagia screening and assessment. Nevertheless, the data were obtained from registered databases, and it was possible that disparities in screening personnel, tools, and time may have influenced the accuracy of the results.

The implementation of clustering analysis on references employing CiteSpace yielded a total of 67 clusters with modularity (Q) and weighted mean silhouette (S) values of 0.8744 and 0.9514, respectively. It indicates that the result of the clusters was convincing. Figure 9B visually displays the 16 largest clusters, with the top four clusters labeled by meta-analysis, prognosis, pneumonia, and VFSS. Clusters #4 rehabilitation, #10 NMES, #11 acupuncture, and #15 electrical pharyngeal stimulation are concentrated on the theme of rehabilitation, while clusters #5 nasogastric tube feeding, #8 feeding route, and #13 nutritional status mainly concern nutritional management.

Reference burst indicates that relevant articles acquire the attention of scholars during a certain timeframe in the research field. As displayed in Figure 9C, Arnold et al. (2016) exhibited the second strongest strength in the analysis of reference bursts, as well as obtaining the second highest co-cited counts. The article by Arnold in 2016 indicated that stroke severity was identified as a significant risk factor for dysphagia. Moreover, deglutition disorder could contribute to increased medical costs due to the requirement for more frequent chest X-rays and antibiotics, prolonged hospital stays and rehabilitation, and a higher risk of pneumonia (Arnold et al., 2016).

## 4. Discussion

### 4.1. Summary of basic information

Over the past two decades, PSD has drawn more attention from researchers, possibly due to its association with higher rates of morbidity and mortality for complications such as aspiration, pneumonia, and malnutrition. Moreover, it imposes a tremendous economic burden and severely affects the quality of life of patients. Bibliometrics analysis displays results in the form of network visualization maps, enabling researchers to efficiently and intuitively understand the research conditions of the topic. This study serves as the first bibliometric analysis of PSD studies, aiming at providing useful insights and guiding future research through the analysis and synthesis of previous relevant literature. Based on a quantitative analysis of the publications over the past two decades, we noticed that the number of published articles increased with fluctuating growth and reached a peak of 137 articles in 2022. In terms of the core author analysis, there were three major collaboration networks headed by Dziewas R, Hamdy S, and Clave P (see Figure 3B). Besides, it was worth noting that Bath and Hamdy acquired the highest average citations, suggesting that their articles were influential and accepted by other scholars. Bath primarily focused on studies of rehabilitation therapies for treating PSD. And Hamdy S conducted studies that encompassed the exploration of central and peripheral nerve stimulation mechanisms, the investigation of sensorimotor mechanisms underlying aspiration, and the observation of swallowing neural networks in PSD patients utilizing functional magnetic resonance imaging (Power et al., 2007; Michou et al., 2014; Mihai et al., 2016).

Among the 58 countries related to this study, China published the most literature, followed by the USA and South Korea. Figure 4A displays the relatively stable cooperative groups led by England, the USA, and Italy. Moreover, England is located in the center of the map, indicating that it has formed close international relationships. International cooperation helps to enhance recognition and influence. Therefore, China should put more emphasis on international collaboration in future research practices. Regarding institutions, the University of Manchester published the most papers, and three of the top five institutions were affiliated with China. This observation demonstrates that China has made significant contributions to PSD research. Interestingly, we observe that these top 10 organizations are universities and research institutions instead of hospitals.

*Dysphagia, Journal of Stroke & Cerebrovascular Diseases*, and *Stroke* rank the top three in terms of publication counts in our journal analysis and have acquired a large number of citations in the analysis of co-cited journals. These journals are influential and have made substantial contributions to the development of PSD research. Our findings indicate that *Stroke* and *Archives of Physical Medicine and Rehabilitation* are categorized within the JCR Q1 partition among the top 10 journals with an impact factor (IF) of 8.3 and 4.3, respectively. Regarding the top 10 co-cited journals, more than half of them were classified as Q1 partitions, with the *Lancet* having the most prominent IF (168.9). It indicates that highly influential journals also pay attention to research on PSD, which may serve as a certain reference for scholars when selecting journals for publication.

### 4.2. Research hotspots

Through the co-occurrence and clustering analysis of keywords as well as co-cited references, we can explore research hotspots in the following aspects.

#### 4.2.1. Screening and assessment of dysphagia in stroke patients

Screening and assessment of dysphagia should be emphasized in stroke units due to the complications and adverse outcomes associated with PSD. Research has demonstrated that delays in screening and comprehensive dysphagia assessment are correlated with an increased risk of post-stroke pneumonia (Bray et al., 2017), a significant cause of death from acute stroke. Consequently, the guideline advocated that dysphagia screening be implemented as soon as possible upon admission (Dziewas et al., 2021), serving various purposes such as evaluating the risk of aspiration, determining the suitability of oral feeding and medications, and reducing the dependence of stroke patients. Dysphagia screening can be effectively performed through the application of standardized screening tests by healthcare professionals who have received specialized training (Rommel and Hamdy, 2016). Regarding the bedside swallow screening methods, a meta-analysis revealed that the Bedside Aspiration Test (water-swallow and oxygen saturation tests), Gugging Swallowing Screen (swallowing tests with water and other consistency foods), and Toronto Bedside Swallowing Screening Test (only water-swallow tests) had a low risk of bias to detect the aspiration risk related to dysphagia (Boaden et al., 2021), which may provide certain references for clinical decision-making.

Guidelines suggested that stroke patients who failed the dysphagia screening or had other clinical predictors of PSD should perform an earlier dysphagia assessment (Wirth et al., 2013; Burgos et al., 2018; Dziewas et al., 2021). Clinical swallowing assessment (CSA) aims to evaluate the risk of aspiration and severity of swallowing impairment, thereby guiding subsequent treatment and instrumental assessment. CSA primarily involves medical history collection, medical and oral examinations, as well as food intake assessment (Rommel and Hamdy, 2016). VFSS and FEES, two instrumental dysphagia assessments, have been shown to enhance the diagnostic accuracy of PSD. VFSS dynamically observes the anatomy and function of the oropharynx and upper esophageal sphincter. It was considered the gold standard for diagnosing oropharyngeal dysphagia and could detect aspiration with or without a cough reflex (Wirth et al., 2016; Helliwell et al., 2023). The penetration-aspiration scale is widely used to evaluate the occurrence and severity of aspiration and penetration in the process of swallowing (Rosenbek et al., 1996). Therefore, early implementation of VFSS not only helps to prevent the occurrence of aspiration pneumonia but also contributes to the efficient management of PSD. However, the application of VFSS may be limited in cases where patients have difficulty maintaining a sitting position or appear to have poor consciousness (Kim et al., 2018). FEES applies a flexible laryngoscope to examine the pharyngeal and laryngeal structures during the swallowing process, and it helps identify pharyngeal residues, penetration, and aspiration (Warnecke et al., 2009; Cosentino et al., 2022). FEES has high sensitivity and can be implemented repeatedly to monitor the changes in swallowing function during the treatment process. Furthermore, it avoids radiation exposure and can be performed at the patient’s bedside (Noordally et al., 2011). However, it is considered an invasive operation, and some patients have described discomfort during the insertion of an endoscope (Kamarunas et al., 2014). Therefore, clinicians are supposed to take into account the overall condition and willingness of the patients to determine the instrumental assessment approaches.

#### 4.2.2. Prevention of pneumonia in patients with PSD

Aspiration of ingested foods, liquids, or oral secretions is considered the primary risk factor for developing pneumonia in stroke patients (Cohen et al., 2016). It was illustrated that stroke patients with dysphagia presented a more than three-fold increase in the risk of developing pneumonia, and an 11-fold increase in the risk appeared in patients with confirmed aspiration (Martino et al., 2005). Therefore, it is necessary to detect dysphagia promptly and strengthen dietary management and stroke care among PSD patients. And it aims to reduce the risk of aspiration, prevent the occurrence of pneumonia, and improve the prognosis of patients. The implementation of texture-modified foods and thickened liquids could alter the properties of both food and liquids, making swallowing safer for patients. Studies have suggested that the thickened liquid decelerates bolus movement and augments its cohesion, thereby reducing the risk of airway penetration and aspiration (Clavé et al., 2006; Steele et al., 2015; Vilardell et al., 2016). Stroke patients are reported to have worse oral hygiene, and systematic implementation of oral hygiene care was associated with an obvious reduction in the risk of stroke-associated pneumonia (Kuo, 2015; Wagner et al., 2016). In terms of drugs, angiotensin-converting enzyme inhibitors play a protective role in reducing the risk of pneumonia. This effect is attributed to the increased substance P and bradykinin levels, thereby enhancing the cough reflex and preventing aspiration (Liu et al., 2012). Besides, a study published in Lancet revealed that it was not recommended to apply antibiotic prophylaxis to prevent the occurrence of post-stroke pneumonia among patients managed in stroke units (Kalra et al., 2015). Hence, screening and assessment of dysphagia, dietary management, oral care, and early interventions should be emphasized in the prevention of pneumonia.

#### 4.2.3. Nutritional management for PSD

PSD poses a challenge for stroke patients to adequately intake fluids and nutrition, which could lead to the occurrence of malnutrition and dehydration (Sabbouh and Torbey, 2018). Studies have pointed out that malnutrition before and after acute stroke leads to increased hospital stays, medical expenses, and mortality rates, as well as poor functional status (Dennis et al., 2005; Kim et al., 2015; Gomes et al., 2016). Hence, it is necessary to implement nutritional screening for stroke patients, facilitating the determination of nutritional support requirements. Nutritional risk screening (NRS 2002), an objective malnutrition screening approach based on a retrospective analysis of nutritional characteristics and clinical results. It discerns malnutrition and predicts the risk of malnutrition by evaluating the indicators of nutrition status, including body mass index, recent weight loss percentage, and changes in dietary intake, in conjunction with an evaluation of disease severity (Kondrup et al., 2003). In addition, the Malnutrition Universal Screening Tool (MUST) is a useful method that was primarily developed for identifying malnutrition in communities. Its use has been extended to other healthcare institutions, such as hospitals, subsequently (Rabito et al., 2017). Enteral tube feeding is an appropriate choice for individuals with PSD who require nutritional support but are unable to obtain sufficient nutrition through oral consumption. According to the released guidelines, the nasogastric tube (NGT) will be recommended for enteral feeding in cases where oral intake is unlikely to be restored within 7 days, while percutaneous endoscopic gastrostomy (PEG) is considered when swallowing dysfunction will remain for more than 4 weeks (Stroud et al., 2003; Wirth et al., 2013; Galovic et al., 2019). The feed or ordinary diet (FOOD) trials indicated that initiating tube feeding within 7 days of admission was related to a 5.8% decrease in the risk of mortality. Moreover, NGT has been shown to achieve better functional outcomes compared to PEG feeding during the first 2–3 weeks following acute stroke (Dennis et al., 2006). Nevertheless, the efficacy of NGT feeding may be compromised due to the frequent occurrence of tube dislodgement or accidental removal. Studies indicated that looped NGT is beneficial in improving the nutritional delivery of patients with PSD and reducing the frequency of reinsertion of NGT (Beavan et al., 2010). As a consequence, applying earlier nutritional screening and appropriate nutritional support approaches may improve physical status and promote rehabilitation following stroke.

#### 4.2.4. Rehabilitation approaches of PSD

Cortical reorganization and neuroplasticity begin in the early stages of stroke; hence, early implementation of rehabilitation is expected to improve the prognosis for stroke patients (Coleman et al., 2017). Rehabilitation exercise can promote long-term improvement of swallowing function through neuroplasticity. These exercises encompass various categories, including oral exercises, indirect swallowing exercises for reinforcing suprahyoid muscles (e.g., chin tuck against resistance and shaker exercise), as well as direct pharyngeal exercises like the effortful swallow and Mendelsohn maneuver (Choy et al., 2023a,b). Besides, neurostimulation techniques including PES, NMES, rTMS, and transcranial direct current stimulation (tDCS) are also exerting significant impacts on the rehabilitation of PSD. PES is a neuromodulation device shown to enhance the reorganization of the motor cortex related to swallowing and promote activation of corticobulbar pathways. It was reported to be safe and effective in enhancing airway protection and swallowing function and contributed to a higher decannulation rate for patients with severe PSD who had undergone a tracheotomy (Dziewas et al., 2018). NMES applies electrical pulses to stimulate muscle contraction, thereby improving and restoring the function of the stimulated muscles (Doan et al., 2022). A meta-analysis manifested that the combination of NMES and swallowing therapy could increase the forward and upward movement distance of the hyoid bone, promote the recovery of swallowing function, and enhance the overall quality of life in PSD patients (Wang Y. et al., 2023). In addition, tDCS and rTMS are two noninvasive methods directly targeting the cerebral cortex. rTMS applies a sequence of continuous stimuli at varying frequencies and sequence intervals to cortical regions (Fisicaro et al., 2019). It effectively modulates the excitability of the targeted cortex area, and its effects still persist even after the completion of the treatment session (Fisicaro et al., 2019). Du et al. (2016) revealed that performing the 3-Hz rTMS on the affected hemisphere or the 1-Hz rTMS on the unaffected hemisphere for five daily sessions promotes the recovery of dysphagia, and the cumulative effect lasted for at least 3 months. tDCS applies a low-intensity current (typically 1–2 mA) to the targeted brain regions, thus regulating neuronal excitability by modifying membrane polarization (Longo et al., 2022). Anodal stimulation increases cortical excitability through neuronal depolarization, whereas cathodal stimulation decreases it through hyperpolarization (Simons and Hamdy, 2017; Li et al., 2021). Wang L. et al. (2023) revealed that routine rehabilitation training and 1 mA anodal tDCS on the swallowing sensory motor cortex on the unlesioned hemisphere can not only alleviate swallowing disorders with long-term efficacy but also decrease infection levels and improve nutritional status.

Acupuncture, a representative and popular method of traditional Chinese medicine, is also a research hotspot, as shown in Figure 9B. A guideline suggests that acupuncture can also be applied to restore swallowing ability in PSD patients (Dziewas et al., 2021). Yao et al. (2023) demonstrated that electroacupuncture at the Lianquan (CV23) acupoint could activate the inputs of the motor cortex to the nucleus tractus solitarii through the parabrachial nuclei, thereby enhancing the function of deglutition in PSD mice. However, it still requires high-level evidence to validate which rehabilitation approach or combinations are preferred to utilize in different phases and lesion locations of stroke.

### 4.3. Future research trends

The latest citation bursts of keywords enable us to effectively capture research frontiers. It was observed that systematic review occurred most recently in the lists of keyword bursts (see Figure 7). In addition, as depicted in Figure 6B, the recent research topic mainly encompasses meta-analysis, noninvasive brain stimulation, and lesion location, which may also provide information for predicting future research directions.

Systematic reviews represent a type of literature review that employs systematic and explicit approaches to identify and analyze relevant studies, resulting in robust conclusions with less subjectivity and bias. It is beneficial for readers to quickly discern the focus of the field and the deficiencies of the previous studies and then direct future research (Siddaway et al., 2019). Meta-analysis is a quantitative research methodology that applies statistical methods to synthesize data from retrieved studies to elucidate a specific research question (Arya et al., 2020). Researchers often integrate the two methods for in-depth analysis and evaluation of publications. When studies focus on distinct treatment protocols within the same area and sometimes even yield contradictory conclusions, it may induce perplexity among readers. Therefore, systematic reviews and meta-analyses regarding the management of PSD are required to better summarize clinical evidence for guiding clinical practice and the development of guidelines.

Noninvasive brain stimulation (NIBS) techniques modulate cerebral activity and excitability through electric or magnetic fields, inducing long-term changes in synaptic plasticity (Fregni and Pascual-Leone, 2007). tDCS and rTMS are regarded as two promising techniques to facilitate the recovery of stroke. The efficacy of these techniques is largely dependent on intensity or frequency, duration, and targeted regions of stimulation. Nonetheless, the selection of these parameters and their impact on clinical outcomes have not yet reached a consensus. He et al. (2022) proposed that high-intensity anodal stimulation at 1.6–2 mA showed more advantages in improving dysphagia than low-intensity stimulation at 1–1.5 mA. However, another meta-analysis reported that lower current intensities were related to better results for PSD (Marchina et al., 2021). Additionally, Xie et al. (2023) suggested that anodal tDCS at 1.4 mA for a duration of 20 min may be a more optimal parameter for PSD compared with the intensities of 1.2, 1.4, 1.5, 1.6, and 2 mA. Regarding the stimulating site, it was reported that conducting tDCS on the bilateral hemispheres might have a superior effect than the unlesioned hemisphere in promoting deglutition recovery (He et al., 2022). However, further exploration is required to elucidate the comparative efficacy between stimulation of the affected and unaffected hemispheres (Marchina et al., 2021).

In terms of rTMS, high-frequency stimulation (>1 Hz) of rTMS increases the cortical excitability, while low-frequency stimulation (≤1 Hz) exerts the suppressive effect (Fitzgerald et al., 2006; Fisicaro et al., 2019). Through subgroup analysis, Xie et al. (2022) indicated that low-frequency rTMS was more effective than high-frequency rTMS in enhancing swallowing capabilities, and applying rTMS to the bilateral and contralateral hemispheres showed significant effectiveness. Nevertheless, Liao et al. (2017) expressed an opposite viewpoint regarding the comparative advantages of high-frequency and low-frequency rTMS. In addition, a study with a recent citation burst (see Figure 9C) found that 1-Hz rTMS combined with swallowing exercises enhanced quality of life but not significantly improved deglutition disorders when compared with swallowing exercises alone (Ünlüer et al., 2019). Due to various confounding factors, there is some divergence in interpreting the efficacy of different parameters of NIBS. Hence, more efforts are required to discern the severity and lesion characteristics of stroke patients and elucidate the recovery mechanism of PSD, thereby optimizing the treatment scheme.

Swallowing involves a series of rapid and highly coordinated neuromuscular movements that depend on the neural network of the cerebral cortex, subcortical structures, and brainstem. Different lesion locations could affect the incidence, severity, and recovery of PSD. A meta-analysis demonstrated that the incidence of swallowing dysfunction after pontine and medulla lesions (especially lateral medulla lesions) was higher compared to cerebellar or midbrain lesions (Flowers et al., 2011). Hamdy indicated that the cortical control of the pharynx represented interhemispheric asymmetry, and acute ischemic stroke in the right hemisphere could cause more severe pharyngeal dysfunction (Hamdy et al., 1998; Wilmskoetter et al., 2018). In addition, the insular cortex is a commonly affected area and may be associated with aspiration (Qiao et al., 2022). The recovery of oral intake could be affected by white matter lesions within the first week, whereas the dysphagia recovery was influenced by lesions in specific cortical nodes of swallowing during the 2–4 weeks (Galovic et al., 2017). Furthermore, studies have also shown a correlation between lesion locations and the occurrence of post-stroke depression. Wei et al. (2015) pointed out that the risk of depression was associated with right hemisphere strokes within 1–6 months after a stroke. Employing vector regression lesion-symptom mapping, a study has indicated that damage to the right insular cortex, right putamen, and inferior frontal gyrus may result in depression symptoms (Klingbeil et al., 2023). Conversely, Zhang et al. (2017) found that left hemisphere strokes appear to be more predisposed to depression among subacute stroke patients. A recent investigation proposed that impaired connections between lesion regions and the left dorsolateral prefrontal cortex in the depression circuit could contribute to depression following stroke (Fan et al., 2023). Consequently, the identification of lesion locations might provide guidance for early screening and subsequent interventions, thereby reducing unfavorable outcomes.

### 4.4. Study limitations

Despite our comprehensive exploration of collaborative and co-occurrence relationships among authors, institutions, countries, and journals, as well as the research hotspots and trends of PSD through the analysis of retrieved articles and their references, there still exist certain limitations. Firstly, to ensure the quality of the retrieved publications and the integrity of the exacted information, we only searched the core collection of Web of Science, potentially omitting important literature available in other databases; Secondly, our inclusion criteria were restricted to publications in English, thus neglecting articles concerning PSD composed in other languages; Thirdly, there exists the possibility of underestimating the academic influence of recently published papers due to their limited citations.

## 5. Conclusion

To our knowledge, this is the first bibliometric analysis of post-stroke dysphagia, presenting the findings in the form of visualized maps and conducting a comprehensive analysis. Our study indicates that there has been a continuous increase in publications on PSD over the past two decades. The most frequently published authors, institutions, countries, and journals are Dziewas R, the University of Manchester, China, and *Dysphagia*, respectively. By analyzing the references, the co-cited journal and author are *Stroke* and Martino R. Moreover, the research hotspots for PSD mainly consist of screening and assessment, prevention of pneumonia, rehabilitation approaches, and nutritional management of PSD. For future research pertaining to PSD, more articles are expected to focus on the topics of systematic review and meta-analysis, noninvasive brain stimulation, and lesion location. In conclusion, our analysis provides scholars with valuable information about PSD and serves as a reference for conducting further exploration in this field.

## Data availability statement

The original contributions presented in the study are included in the article/supplementary material, further inquiries can be directed to the corresponding author.

## Author contributions

FX: Conceptualization, Data curation, Formal analysis, Writing – original draft. LB: Data curation, Formal analysis, Writing – review & editing. ZD: Data curation, Formal analysis, Software, Writing – review & editing. HC: Conceptualization, Funding acquisition, Supervision, Writing – review & editing.
